# Posttraumatic Growth in Intensive Care Unit Health Care Professionals After COVID-19

**DOI:** 10.1001/jamanetworkopen.2025.27443

**Published:** 2025-08-25

**Authors:** Élie Azoulay, Laurent Argaud, Vincent Labbé, Guillaume Dumas, Fabrice Bruneel, Mercé Jourdain, Christophe Guitton, Amélie Seguin, Samir Jaber, David Schnell, Isabelle Vinatier, Fanny Ardisson, Michel Ramakers, Antoine Herault, Olivier Lesieur, Alain Cariou, Antoine Vieillard-Baron, Olivier Guisset, Frédéric Pochard, Michael Darmon, Nancy Kentish-Barnes

**Affiliations:** 1 Medical Intensive Care Unit, Assistance Publique–Hôpitaux de Paris, Saint-Louis Hospital, Paris-Cité University, Paris, France, INSERM UMR1342 Institut de Recherche Saint-Louis; 2 Medical Intensive Care Unit, Edouard Herriot Hospital, Hospices Civils de Lyon, Lyon, France; 3 Intensive Care Department, Hôpital Universitaire de Bruxelles, Université Libre de Bruxelles, Brussels, Belgium; 4 Service de Médecine Intensive-Réanimation, CHU Grenoble-Alpes, Université Grenoble-Alpes, INSERM U1300-HP2, Paris, France; 5 Medical-Surgical Intensive Care Unit, Versailles Center, Mignot Hospital, Le Chesnay, France; 6 Intensive Care Unit, Lille University Hospital–Roger Salengro Site, INSERM, Lille, France; 7 Service de Réanimation Médico–Chirurgicale Polyvalente, Centre Hospitalier Le Mans, Le Mans, France; 8 Medical Intensive Care Unit, UR 4334 Movement-Interactions-Performance Research Unit, Nantes University Hospital, Nantes, France; 9 Department of Anesthesiology and Critical Care Medicine B (DAR B), Saint-Eloi Hospital, University Teaching Hospital of Montpellier, Montpellier, France; 10 CH Angoulême, Médecine Intensive Réanimation, Angoulême, France; 11 Service de Réanimation, Centre Hospitalier Départemental de la Vendée, La Roche sur Yon, France; 12 Intensive Care, University Hospital of Guadeloupe, Pointe-à-Pitre, Les Abymes, French West Indies, France; 13 Service de Médecine Intensive Réanimation, Centre Hospitalier Mémorial de Saint-Lô, Saint-Lô, France; 14 Medical Intensive Care Unit, Rouen University Hospital, Rouen, France; 15 Médecine Intensive Réanimation, CH La Rochelle, La Rochelle, France; 16 Cochin University Hospital, Assistance Publique–Hôpitaux de Paris, Paris-Cité University, Paris, France; 17 Assistance Publique–Hôpitaux de Paris, Hospital Ambroise Pare, Boulogne, France, INSERM UMR-1018, CESP, Team Kidney and Heart, University of Versailles Saint-Quentin en Yvelines, Villejuif, France; 18 Medical Intensive Care Unit, Saint-André Hospital, Bordeaux, France

## Abstract

**Importance:**

Posttraumatic growth (PTG) refers to positive psychological changes following adversity, including deeper relationships and a greater appreciation for life.

**Objective:**

To assess PTG among intensive care unit (ICU) health care professionals 4 years after the COVID-19 pandemic and explore its association with resilience, anxiety, and depression.

**Design, Setting, and Participants:**

This cross-sectional study invited ICU health care professionals (nursing staff, medical staff [residents, interns, clinical fellows, and senior intensivists], and other professionals providing patient care) in ICUs in general or university-affiliated hospitals in France and Belgium to complete online questionnaires between March 15 and May 15, 2024.

**Exposures:**

PTG, resilience, anxiety, and depression.

**Main Outcomes and Measures:**

The primary outcome was PTG, and secondary outcomes were anxiety, depression, and resilience. Participants completed validated self-reported questionnaires, including the Posttraumatic Growth Inventory (PTGI), Hospital Anxiety and Depression Scale (HADS), and 10-item Connor-Davidson Resilience Scale (CD-RISC 10). Visual analog scales (VASs) assessed professional experiences and psychological impact. Multivariate linear regression identified factors associated with PTG.

**Results:**

Among 1371 health care professionals in 23 ICUs, 850 (62%) responded (median age, 39 years [IQR, 32-46 years]; 574 [68%] women). The median PTGI score was 50 (IQR, 33-64), with nursing staff reporting higher PTG than medical staff (51 [IQR, 34-65] vs 47 [IQR, 28-61]; *P* = .02), mainly in personal strength, spiritual change, and appreciation of life. Anxiety and depression symptoms were present in 492 respondents (58%) and 219 respondents (26%), respectively. Lower PTG was associated with psychological fatigue (regression coefficient, 1.43; 95% CI, 0.91-1.96; *P* < .001), ICU conflicts (regression coefficient, 0.62; 95% CI, 0.05-1.19; *P* = .03), and perceived deterioration in family-centered care (regression coefficient, −7.47; 95% CI, −1.10 to −13.80; *P* = .02). Higher PTG was correlated with higher resilience (Spearman correlation coefficient, 0.24; 95% CI, 0.17-0.30; *P* < .001) and was associated with a change in personal life since the pandemic (regression coefficient, 1.80 [95% CI, 1.13-2.47] per VAS point; *P* < .001).

**Conclusions and Relevance:**

In this cross-sectional study performed 4 years after the start of the COVID-19 pandemic, ICU health care professionals, particularly nursing staff, exhibited significant PTG. Resilience, rather than psychological distress, emerged as a key driver of PTG, and deterioration in family-centered care was a major contributing factor, underscoring the need for targeted well-being and resilience-building strategies to enhance health care professionals’ mental health and professional fulfillment while also improving patient and family care.

## Key Points

**Question:**

Did health care professionals in intensive care units (ICUs) during the COVID-19 pandemic experience posttraumatic growth (PTG)?

**Findings:**

In this cross-sectional study of 850 health care professionals conducted 4 years after the start of the COVID-19 pandemic, nursing staff reported higher PTG than medical staff. Lower PTG was associated with psychological fatigue, ICU conflicts, and a decline in family-centered care, and higher PTG correlated with greater resilience and personal life changes since the pandemic.

**Meaning:**

The findings suggest that resilience, rather than psychological distress, is a key driver of PTG, highlighting the need for targeted well-being and resilience-building strategies.

## Teaser

This cross-sectional study investigates positive psychological changes after adversity, known as posttraumatic growth, in health care professionals who worked in intensive care units (ICUs) during the COVID-19 pandemic in France and Belgium.

## Introduction

Posttraumatic growth (PTG) refers to the positive psychological changes that individuals may experience as a result of struggling with highly challenging, stressful, or traumatic events.^1,2^ PTG includes improvements in areas such as personal strength, appreciation for life, relationships with others, new possibilities in life, and spiritual development.^3^ PTG is not about returning to a previous state of well-being but rather about achieving a higher level of functioning and a deeper understanding of oneself and the world.^4^ In the context of intensive care unit (ICU) health care professionals after the COVID-19 pandemic, PTG can manifest as an enhanced sense of personal resilience, deeper connections with colleagues and patients, a greater sense of purpose and fulfillment in work, and an increased appreciation for life and its fragility.^5^ Recognizing and fostering PTG can be crucial for the long-term mental health and well-being of health care professionals who have faced extraordinary pressures and challenges.

While resilience and PTG are often discussed in similar contexts, they represent distinct psychological processes.^6,7^ *Resilience* refers to an individual’s ability to adapt to adversity, maintaining relatively stable levels of psychological functioning despite stress or trauma. In contrast, PTG goes beyond simple recovery; it involves a profound transformation in which individuals experience positive psychological changes as a result of struggling with adversity. This can manifest as an enhanced appreciation for life, stronger relationships, or a redefined sense of purpose. In the context of intensive care, understanding this distinction is crucial, as interventions may aim to foster not only resilience but also PTG in health care professionals.^8^

PTG in ICU health care professionals after the COVID-19 pandemic has become a significant area of research interest.^5^ These individuals faced unprecedented challenges, including high patient mortality, extended shifts, and the constant risk of infection. Despite the immense stress and trauma, many ICU health care professionals have reported positive psychological changes.^9^ Assessing PTG in these professionals after the pandemic can inform strategies to bolster the mental health and well-being of ICU health care professionals in future crises. For instance, whether PTG may highlight the resilience and adaptability of health care professionals remains uncertain.^6^ The insights gained from studying PTG could underscore the importance of providing adequate psychological support and resources to help health care professionals navigate and grow from their experiences.^10,11^

## Methods

The Comité de Protection des Personnes Sud-Méditerranée ethics committee approved this cross-sectional study. This was the fourth cross-sectional study conducted by our group during and after the COVID-19 pandemic (each of the other 3 studies covered different outbreaks).^12,13,14^ This study received a waiver of informed consent from the ethics committee because no patients or patients’ relatives were included. All health care professionals working in participating ICUs from general or university-affiliated hospitals in France and Belgium were eligible to participate in the study on a voluntary basis. An invitation to take part in this cross-sectional study was sent to all health care professionals working in the participating ICUs, using mailing lists, groups from a messaging app, a poster with QR codes in each ICU, and local invitations by study investigators. Health care professionals were defined as nursing staff members (nurses and nursing assistants), medical staff members (residents, interns, clinical fellows, and senior intensivists), and other professionals providing patient care in the ICUs. The questionnaire (eAppendix in Supplement 1) was made available online between March 15 and May 15, 2024, to each of the health care professionals who worked in the participating ICUs.^12,13^ At the time of this study, no patient with COVID-19 was being treated in the participating ICUs. This study is reported in accordance with the Strengthening the Reporting of Observational Studies in Epidemiology (STROBE) reporting guideline.^15^

Data reported in tables and figures were collected prospectively. The survey items have been identified from the literature^5^ and previous studies from our group.^12,13^ Exposure to COVID-19 was assessed using the number of patients managed. Health care professionals’ characteristics were recorded at the end of the survey.

The survey was designed to capture post–COVID-19 experience. It included 3 validated, self-reported questionnaires assessing PTG, anxiety and depression, and resilience, respectively. Several psychometric tools have been developed to assess individuals’ ability to initiate positive change after adversity. The most widely used and validated tool is the Posttraumatic Growth Inventory (PTGI), an individual self-assessment scale developed by Tedeschi and Calhoun,^16^ which assesses PTG multidimensionally, with a validated French version by Lelorain et al.^17^ The PTGI evaluates 5 key dimensions of PTG, namely appreciation of life (deeper gratitude for everyday experiences and a renewed sense of luck to be alive), relationships with others (stronger, more intimate and meaningful connections), personal strength (increased resilience, confidence, and ability to handle adversity), new possibilities (behaviors and life changes initiated or catalyzed by the crisis), and spiritual growth (enhanced spirituality or existential reflection). These 5 domains were identified through factor analysis based on items developed from interviews and literature reviews on PTG.^16^ Each of the 21 items is rated on a scale from 0 (no positive change experienced) to 5 (significant positive change experienced) to best capture the individual’s growth. The sum of the ratings for the 21 items provides a global score ranging from 0 to 105, with higher scores indicating greater PTG.^18^ Symptoms of anxiety and depression were assessed using the Hospital Anxiety and Depression Scale (HADS),^12^ with scores greater than 7 of 21 on the relevant subscale indicating the presence of symptoms of anxiety or depression. Resilience was assessed using the unidimensional 10-item Connor-Davidson Resilience Scale (CD-RISC 10), which measures the ability to adapt positively to adversity.^13,19^ This tool evaluates key aspects such as adaptability, personal competence, social support, emotional regulation, stress control, spiritual influence, acceptance, determination, decision-making, and sense of purpose. Each of the 10 items is rated on a 0 to 4 Likert scale (0 indicating “not true at all” and 4, “true nearly all the time”), yielding a total score from 0 to 40, with higher scores indicating greater resilience.

For variables depicting the COVID-19 experience, the responses were either binary (“yes” or “no”) or made on visual analog scales (VASs) with scores ranging from 0 (no symptom) to 10 (the most intense symptom) and assessed frustration, fear, isolation, pride, exhaustion, fatigue, motivation, commitment to work, support, sense of gratification, and need for change. VASs are easy and rapid to complete and have been proved reliable for measuring characteristics, subjective phenomena, and attitudes that are expected to range across a continuum of values and for which direct measurements cannot be readily obtained.^20^ The COVID-19 infodemic was scored on a VAS ranging from 0 (no misinformation) to 10 (overwhelming misinformation). The infodemic refers to the rapid spread of both accurate and inaccurate information, blending facts with rumors and fears, which can fuel confusion, risky behaviors, and mistrust in health authorities, ultimately weakening the public health response.^21^

### Study Outcomes

PTG was the primary outcome and was measured using the PTGI. We did not use a cutoff value for this score. The secondary outcomes were symptoms of anxiety and depression, defined as scores greater than 7 on the relevant HADS subscales, and resilience, measured using the CD-RISC-10 score. We did not use a cutoff value for the CD-RISC-10 score.

### Statistical Analysis

The data are described as median (IQR) or number (percentage). Categorical variables were compared using the Fisher exact test and continuous variables using the nonparametric Wilcoxon test or Kruskal-Wallis test. Spearman coefficients were computed to assess correlations.

Factors independently associated with PTGI or with CD-RISC 10 score were identified by building a linear regression model. Logistic regression was also performed to assess independent variables associated with symptoms of anxiety and depression. For all models, we first performed univariate analyses including all the variables shown in eTables 1 to 3 in Supplement 1. Variables with *P* values less than .20 for associations were used to build multivariate models. The final models were obtained by stepwise variable selection using an automatic procedure based on the Akaike information criterion. Interactions and correlations between the explanatory variables were carefully checked. For logistic models, continuous variables were checked for log-linearity assumptions and dichotomized if needed. For linear models, the linearity assumption was carefully checked. We assessed calibration and discrimination of multivariate logistic regression models and the percentage of variation (*r*^2^) explained by the model for multivariate linear regression models. Surface plots were created to depict associations between important variables. We did not adjust for multiple comparisons. No imputation methods were used.

All tests were 2 sided, and *P* values less than .05 were considered to indicate statistical significance. Analyses were done using R, version 3.6.2 (R Project for Statistical Computing).

## Results

Of 1371 included health care professionals working in 23 ICUs, 850 (62%) completed the questionnaires (median age, 39 years [IQR, 32-46 years]; 276 [32%] men, 574 [68%] women). Participants included 507 nursing staff (60%), 267 medical staff (31%), and 76 allied health care professionals (9%) (eTable 1 in Supplement 1). Most participants (545 [64%]) worked in university-affiliated hospitals, and 85 (10%) were not working in the ICU during the COVID-19 pandemic. Since the pandemic, extension of visitation policies occurred in 6 ICUs (26%), and 285 respondents (34%) reported that improvement in family-centered care was sustained over time.

Looking back at the pandemic, respondents reported being still very affected by the exhaustion and psychological fatigue they experienced during the pandemic (median VAS exhaustion score, 7 [IQR, 3-8]), by the number of deaths (5 [IQR, 2-7]), and by the suboptimal end-of-life care that was delivered during the surge (6 [IQR, 3-8]). Caregiving during the pandemic continued to be perceived as exhausting (6 [IQR, 4-7]) and frustrating (5 [IQR, 3-7]). Participants also considered the infodemic phenomenon more frequent now than before the pandemic (7 [IQR, 5-9]). Overall, working conditions and public esteem were perceived as unchanged from pre–COVID-19 levels. Respondents rated their professional well-being as very good (7 [IQR, 5-8]) and described their current situation as minimally frightening (3 [IQR, 2-5]), isolating (2 [IQR, 1-4]), or conflict-generating (2 [IQR, 1-4]). Four years after the pandemic onset, they reported that they remained highly engaged (7 [IQR, 5-9]), satisfied with family-centered care (7 [IQR, 5-8]), and proud of their work (8 [IQR, 7-9]). Respondents expressed, however, the need for a major professional change (7 [IQR, 5-9]), including stronger engagement in the ICU and in the hospital (285 respondents [34%]); intention to leave the ICU and pursue career development (285 [34%]); or intention to leave the ICU and the hospital for a complete career change (199 [23%]). Compared with medical staff, nursing staff reported significantly higher VAS scores for certain difficult COVID-19 experiences (eFigure 1 in Supplement 1).

**Figure 1.**
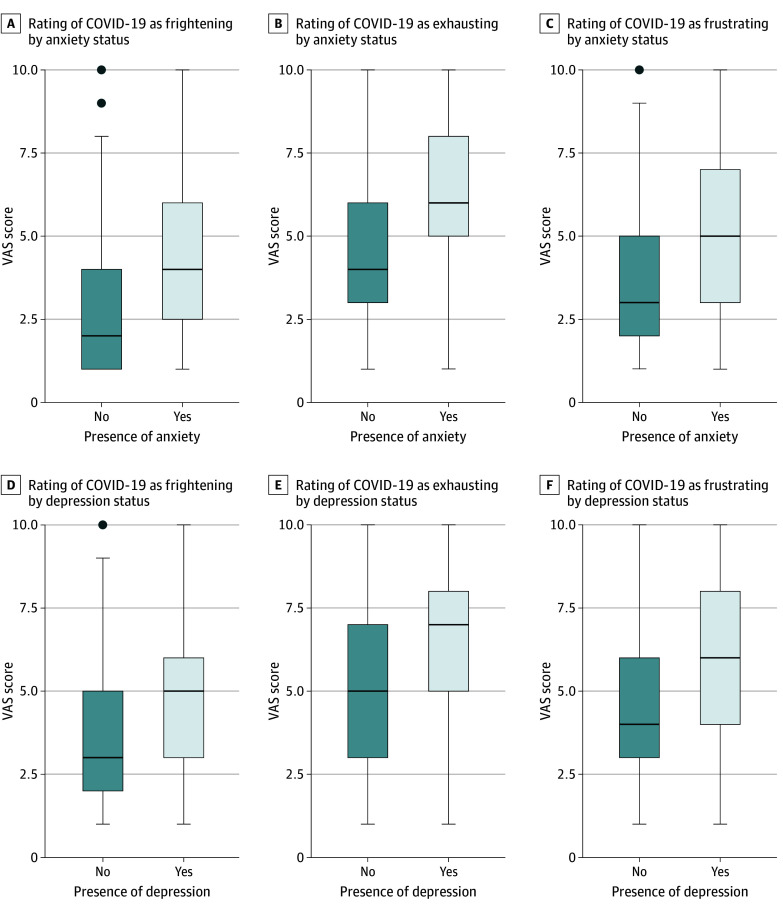
Intensity of Unidimensional Measures in Intensive Care Unit Health Care Professionals With and Without Symptoms of Anxiety, Depression, and Resilience Ratings were in response to the question “Looking back to the pandemic, could you rate if the experience is still frightening, exhausting, frustrating?” and used a visual analog scale (VAS) with 2 anchors: 0 (no symptom) and 10 (most intense symptom). The horizontal bar inside the boxes indicates the median VAS score and the lower and upper ends of the boxes, the first and third quartiles. The whiskers indicate variability outside the upper and lower quartiles, and data more extreme than the whiskers are plotted individually as outliers (shaded circles).

**Figure 2.**
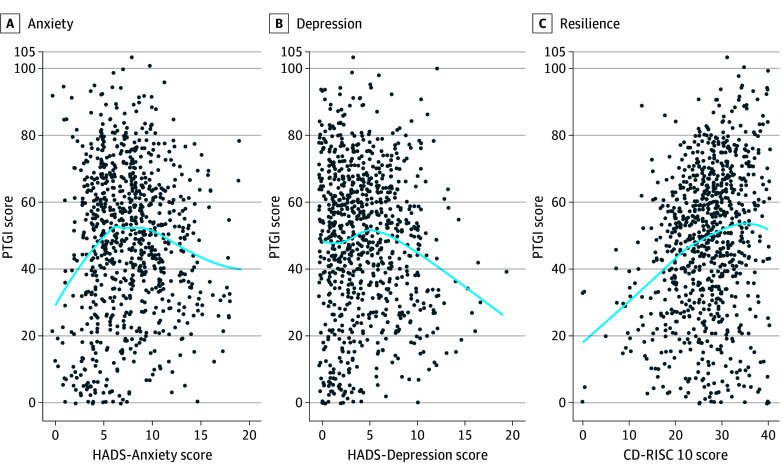
Correlation Between Posttraumatic Growth Inventory (PTGI) Scores and Anxiety, Depression, and Resilience Anxiety was measured with the Hospital Anxiety and Depression Scale (HADS)–Anxiety subscale and depression with the HADS-Depression subscale (scores greater than 7 of 21 on each subscale indicate presence of symptoms of anxiety or depression); resilience was measured with the 10-item Connor-Davidson Resilience Scale (CD-RISC 10; score range, 0-40, with higher scores indicating greater resilience). PTGI scores range from 0 to 105, with higher scores indicating greater PTG. There was a significant positive correlation between PTG and resilience but not between PTG and anxiety or depression.

As shown in Table 1, the median PTGI score was 50 (IQR, 33-64), including 9 (IQR, 6-11) for appreciation of life, 13 (IQR, 8-17) for new possibilities, 15 (IQR, 8-21) for relating to others, 11 (IQR, 7-14) for personal strength, and 2 (IQR, 0-4) for spiritual changes. Of note, the median PTGI score was higher in nursing staff than in medical staff (51 [IQR, 34-65] vs 47 [IQR, 28-61]; *P* = .02), mainly driven by greater personal-strength, spiritual-change, and appreciation-of-life subscale scores. Symptoms of anxiety and depression were found in 492 respondents (58%) and 219 respondents (26%), respectively. The median CD-RISC 10 score was 27 (IQR, 23-31). Anxiety, depression, or resilience scores were not significantly different across ICU roles. Higher VAS scores for certain difficult COVID-19 experiences were reported by health care professionals with symptoms of anxiety, depression, or resilience (Table 2, Figure 1, and eFigure 2 in Supplement 1). As shown in Figure 2, PTG was not correlated with anxiety or depression but was significantly positively correlated with resilience (Spearman correlation coefficient, 0.24; 95% CI, 0.17-0.30; *P* < .001). As shown in eTable 1 in Supplement 1, there was no significant difference in the PTG score across ICU professionals.

**Table 1.** Posttraumatic Growth, Resilience, and Symptoms of Anxiety and Depression

Questionnaire	Score^a^
Posttraumatic Growth Inventory^b^	50 (33-64)
Appreciation of life	9 (6-11)
Relationships with others	15 (8-21)
Personal strength	11 (7-14)
New possibilities	13 (8-17)
Spiritual growth	2 (0-4)
CD-RISC 10^c^	27 (23-31)
Hospital Anxiety and Depression Scale^d^	
Anxiety subscale	7 (5-10)
Health care professionals with symptoms of anxiety, No. (%)	492 (58)
Depression subscale	4 (2-7)
Health care professionals with symptoms of depression, No. (%)	219 (26)

Abbreviation: CD-RISC 10, 10-item Connor-Davidson Resilience Scale.

^a^ Data are presented as median (IQR) score unless otherwise indicated.

^b^ Scores range from 0 to 105, with higher scores indicating greater posttraumatic growth.

^c^ Scores range from 0 to 40, with higher scores indicating greater resilience.

^d^ Scores greater than 7 of 21 indicate presence of symptoms of anxiety or depression.

**Table 2.** Multivariable Analysis of Factors Associated With Symptoms of Anxiety, Depression, and Resilience

Health care professional characteristic or experience	Independent association with higher VAS score, OR (95% CI)		
	Anxiety	Depression	Resilience
Male sex	None	1.40 (0.94-2.09)	None
Age	0.99 (0.97-1.00)	None	None
Experiences reported 4 y postpandemic^a^			
Exhausted	1.16 (1.06-1.28)	1.14 (1.03-1.26)	None
Frustrated	1.16 (1.06-1.28)	None	1.12 (1.02-1.89)
Isolated	None	1.20 (1.10-1.31)	0.88 (0.85-0.98)
Motivated	None	0.83 (0.76-0.90)	1.12 (1.05-1.20)
Proud of the work done	0.88 (0.81-0.97)	None	1.11 (1.02-1.18)
In need of professional change	1.16 (1.09-1.26)	1.14 (1.06-1.22)	None
Psychological fatigue	1.09 (1.03-1.15)	1.17 (1.09-1.26)	None
Concerns with end-of-life care during COVID-19	None	None	0.88 (0.82-0.98)
Time spent reading <30 min/d	1.54 (1.11-2.14)	2.14 (1.45-3.20)	None
CD-RISC 10 items	0.93 (0.91-0.96)	0.96 (0.93-0.99)	None

Abbreviations: CD-RISC 10, 10-item Connor-Davidson Resilience Scale; OR, odds ratio; VAS, visual analog scale.

^a^ A VAS was used to assess the intensity of unidimensional measures. Two anchors were provided to participants: 0 (no symptom) and 10 (the most intense symptom).

There was a significant correlation between PTG and difficulties with end-of-life care during the pandemic (*R*, 0.16; 95% CI, 0.09-0.23). PTGI score was also significantly negatively correlated with increasing age as a continuous variable (*R*, −0.13; 95% CI, −0.06 to −0.19) and with a negative ICU experience (*R*, −0.14; 95% CI, −0.07 to −0.20).

In the multivariable linear regression model (Table 3), factors associated with PTG included reporting that personal life is better as compared with before the pandemic (regression coefficient, 1.80 [95% CI, 1.13-2.47] per VAS point; *P* < .001), that family-centered care did not improve (−7.23; 95% CI, −4.17 to −10.30; *P* < .001), or that family-centered care worsened (−7.47; 95% CI, −1.10 to −13.80; *P* = .02), as well as reporting the advent of a new personal event (4.60; 95% CI, 1.72-7.47; *P* = .003), experiencing psychological fatigue (regression coefficient, 1.43; 95% CI, 0.91-1.96; *P* < .001), and perceiving ICU conflicts (regression coefficient, 0.62; 95% CI, 0.05-1.19; *P* = .03). The CD-RISC 10 score (regression coefficient, 0.85 [95% CI, 0.64-1.06] per score point; *P* < .001) was also associated with PTG.

**Table 3.** Variables Associated With Posttraumatic Growth by Multivariable Linear Regression

Variable	Regression coefficient (95% CI)	*P* value
Family-centered care		
Improved	1 [Reference]	NA
Did not improve	−7.23 (−4.17 to −10.30)	<.001
Worsened	−7.47 (−1.10 to −13.80)	.02
Psychological fatigue	1.43 (0.91 to 1.96)	<.001
Perceived ICU conflict	0.62 (0.05 to 1.19)	.03
Personal life is better compared with before the pandemic, per VAS point	1.80 (1.13 to 2.47)	.001
A new personal event happened since the pandemic	4.60 (1.72 to 7.47)	.003
CD-RISC 10 score, per score point	0.85 (0.64 to 1.06)	<.001

Abbreviations: CD-RISC 10, 10-item Connor-Davidson Resilience Scale; ICU, intensive care unit; NA, not applicable; VAS, visual analog scale.

## Discussion

The COVID-19 pandemic placed unprecedented psychological, emotional, and physical strain on ICU health care professionals,^12,13,22,23^ exposing them to extreme stressors, moral distress, and relentless professional demands. While previous research has extensively documented the psychological toll of the pandemic, including burnout, posttraumatic stress disorder (PTSD), anxiety, and depression,^12,13,14,24,25^ far less attention has been given to the potential for PTG. This cross-sectional study represents, to our knowledge, the first ICU-focused assessment of PTG 4 years after the pandemic onset, offering new insights into the capacity for growth, adaptation, and resilience in critical care settings.

PTG is a distinct psychological phenomenon, representing not a return to baseline but an evolution toward higher functioning and a deeper sense of purpose following adversity.^1,16^ One of the salient findings from this study was the higher PTG observed among nursing staff compared with medical professionals. Nurses often engage in longer, more direct patient interactions, forming stronger emotional bonds and facing greater exposure to suffering and end-of-life care challenges.^26,27^ These experiences may serve as catalysts for reflection, reevaluation of priorities, and deeper existential questioning, ultimately fostering greater PTG. The specific domains where PTG was highest—relating to others, new possibilities, and personal strength—suggest that many ICU nurses emerged from the pandemic with a renewed sense of resilience, purpose, and gratitude.

Despite its well-established psychological framework, PTG has been little explored in post–COVID-19 research, especially in ICU-focused studies. The overwhelming emphasis on negative mental health outcomes has overshadowed opportunities to understand, support, and reinforce positive adaptation mechanisms.^7^ Recognizing PTG in ICU health care professionals is not about minimizing distress but rather broadening the narrative, acknowledging that beyond the suffering, there is also resilience, meaning-making, and transformation.^7,28^

Ignoring PTG in ICU professionals represents a missed opportunity for workforce recovery strategies. Unlike traditional burnout prevention measures, which focus on reducing stress, interventions aimed at fostering growth-oriented coping could proactively strengthen ICU professionals’ mental health.^2,10,11^ These might include structured debriefings, facilitated self-reflection, resilience training, and peer-support programs designed to help health care professionals process trauma in a way that promotes personal and professional growth.

Four years after the pandemic, ICU health care professionals had PTGI levels consistent with those of patients with PTSD.^29^ This result is consistent with the high proportion of professionals who expressed a desire to leave the ICU.^30,31^ This study identified suboptimal end-of-life care and family-centered care during the COVID-19 pandemic as major potential barriers to psychological growth in ICU staff. It is crucial to emphasize the mutual relationship between health care professionals’ well-being and quality of care, both in the ICU^13,32^ and other settings.^33,34^ The COVID-19 pandemic highlighted how the absence of patients’ family members hindered communication and emotional support, exacerbating distress and worsening end-of-life care. These factors were independent predictors of PTSD and burnout among ICU health care professionals.^13,35,36^ Conversely, health care professionals experiencing high levels of burnout and moral distress are less likely to provide focused, compassionate care and effective communication with patients and families.^32,33^ This underscores the bidirectional responsibility in ICU care: preventing burnout is essential for supporting families, just as a well-supported and trained workforce thrives in a high-standard ethical climate that fosters both resilience and quality care.

Our findings have direct implications for mental health strategies for ICU professionals. They highlight the need to move beyond a deficit-focused approach to ICU professionals’ well-being. Instead of solely addressing burnout, PTSD, and distress, health care institutions should consider incorporating PTG-based interventions that empower professionals to reframe adversity as a source of strength and meaning. Strategies to cultivate PTG could include psychological support programs that not only mitigate distress but also encourage self-reflection and personal growth; narrative-based interventions, such as journaling or peer-sharing, to reconstruct difficult experiences into meaningful narratives; and training in meaning-centered coping, helping health care professionals recognize the value in their work and experiences.

### Limitations

This study has limitations. First, we did not assess the trajectory of PTG over time, which could have provided insight into different adaptation patterns among ICU health care professionals. Longitudinal data could help identify subgroups with distinct needs, such as targeted training, psychological support, or communication reinforcement.^37^ Along this line, assessing PTSD in health care professionals would have added to these adaptation patterns. Second, the study included health care professionals with varying levels of COVID-19 exposure and experience. While this heterogeneity might introduce variability, previous research suggests that the number of patients with COVID-19 managed was not directly associated with psychological burden or resilience-building capacity.^12,13^ Therefore, exposure alone may not fully explain differences in PTG outcomes. Third, other unmeasured professional factors emerging 4 years after the start of the COVID-19 pandemic may have influenced PTG trajectories. However, despite these evolving professional and personal dynamics, our findings underscore the persistent pandemic-associated mental health burden on ICU health care professionals, with high rates of fatigue, exhaustion, and distress still reported. Future studies should explore the long-term evolution of PTG, its determinants, and potential interventions to sustain positive adaptation in ICU professionals.

## Conclusions

This cross-sectional study found that 4 years after the start of the COVID-19 pandemic, ICU health care professionals, particularly nursing staff, exhibited substantial PTG, with resilience emerging as a key driver. This study underscores PTG as a crucial yet underused concept in ICU health care professionals’ recovery after COVID-19. While trauma has been extensively studied, fostering growth remains an overlooked but essential aspect of long-term well-being. By integrating PTG into ICU professionals’ recovery strategies, we can not only mitigate distress but actively reinforce professional resilience, job satisfaction, and workforce retention in critical care settings. Future research should systematically assess how PTG can be encouraged and sustained, ensuring that ICU professionals are not only supported in times of crisis but also empowered to emerge stronger.

## Notes

Supplement 1. **eTable 1.** Respondent Characteristics **eTable 2.** PTG Scores Across Professions **eTable 3.** Comparison of Health Care Professionals’ Characteristics According to Whether They Worked in the ICU During the COVID-19 Surges or Not **eFigure 1.** HCPs’ Retrospective Experience of the COVID-19 Pandemic Measured by VAS **eFigure 2.** Intensity of Unidimensional Measures **eAppendix.** Survey Instrument

Supplement 2.
Data Sharing Statement
