# Supplementary material for: Posttraumatic Growth in Intensive Care Unit Health Care Professionals After COVID-19
Source: JAMA Netw Open. 2025 Aug 25;8(8):e2527443. doi: 10.1001/jamanetworkopen.2025.27443 (PMC12379108; doi:10.1001/jamanetworkopen.2025.27443)
Supplement: Supplement 1. — eTable 1. Respondent Characteristics eTable 2. PTG Scores Across Professions eTable 3. Comparison of Health Care Professionals’ Characteristics According to Whether They Worked in the ICU During the COVID-19 Surges or Not eFigure 1. HCPs’ Retrospective Experience of the COVID-19 Pandemic Measured by VAS eFigure 2. Intensity of Unidimensional Measures eAppendix. Survey Instrument [file jamanetwopen-e2527443-s001.pdf]

Supplementary Online Content

Azoulay E, Argaud L, Labbé V, et al. Posttraumatic growth in intensive care unit health care professionals after COVID-19. *JAMA Netw Open*. 2025;8(8):e2527443. doi:10.1001/jamanetworkopen.2025.27443

eTable 1. Respondent Characteristics

eTable 2. PTG Scores Across Professions

eTable 3. Comparison of Health Care Professionals' Characteristics According to Whether They Worked in the ICU During the COVID-19 Surges or Not

eFigure 1. HCPs' Retrospective Experience of the COVID-19 Pandemic Measured by VAS

eFigure 2. Intensity of Unidimensional Measures

eAppendix

This supplementary material has been provided by the authors to give readers additional information about their work.

© 2025 Azoulay E et al. *JAMA Network Open*.

eTable 1: Respondent characteristics

	All respondents, N=850 (%) or Median (IQR)
**Male sex; Age, years**	276 (32.5%); 39 (32-46)
**University affiliated hospitals**	545 (65%)
**Role in the ICU**	
Nurses	367 (43%)
Nurse assistants,	140 (16%)
Attending physicians,	121 (14%)
Fellows,	62 (7%)
Residents,	42 (5%)
Head nurses,	38 (5%)
Tenured university hospital faculty staff,	27 (3%)
Physiotherapists,	22 (3%)
Medical students,	15 (2%)
Psychologists,	9 (1%)
Secretaries	7 (1%)
**Experience in the ICU, months**	76 (36-156)
**Have worked during the pandemic**	765 (90%)
**Received COVID-19 vaccine in 2024**	512 (61%)
**Compared to pre-COVID, respondents reported**	
Use of psychotropic drugs	169 (20%)
Need for a psychological support	224 (26%)
Excessive alcohol intake / cannabis / recreative drugs	158(19%)/72(8%)/49(6%)
**Visitation policies for family members (compared to before the pandemic)**	
Unchanged	524 (62%)
Free visitation policies	117 (14%)
Family visits not allowed	114 (14%)
Did not work during the pandemic	85 (10%)
**Family centered care**	
Unchanged	446 (52%)
Improved	269 (32%)
Worsened	50 (6%)
Unknown	85 (10%)
**Since the onset of the pandemic, I have been supported by**	
Family and friends	782 (92%)
My colleagues	612 (72%)
My superiors	230 (27%)
My institution	34 (4%)
The government	17 (2%)
**My personal life is better compared to before the pandemic**	5 (5-7)
**Today**	
My physical fatigue is	6 (3-8)
My psychological fatigue is	7 (3-8)
My motivation has increased	7 (5-9)
I am proud of the work I have been doing	8 (6-9)
I need professional changes	7 (5-9)
**Daily time on the social media > 30 minutes**	510 (60%)
**Time reading of a book every day > 30 minutes**	510 (60%)

ф Visual analogue scales were used to assess the intensity of unidimensional measures. Two anchors were provided to family members for 0 (no symptom/lowest rating) and 10 (the most intense symptom/highest rating).

ϒ including videoconferences, routine call from healthcare workers to family members and WhatsApp groups

© 2025 Azoulay E et al. *JAMA Network Open*.

eTable 2: PTG scores across professions

PTG score<br>Median (IQR)	Nurses	Physicians	House staff (interns, residents and fellows)	Other professionals	P Value
Men	49 (30-63)	49 (30-64)	46 (29-63)	41 (30-54)	0.71
Women	52 (35-66)	47 (30-60)	61 (51-66)	50 (37-69)	0.22

© 2025 Azoulay E et al. *JAMA Network Open*.

eTable 3: Comparison of health care professionals' characteristics according to whether they worked in the ICU during the COVID-19 surges or not

	Worked in ICU during COVID-19 surges N=765	Did not worked in the ICU during COVID-19 surges; N=85	P Value
**Male sex**	256 (33.5%)	20 (23.5%)	0.005
**Age (years)**	39 (32-47)	29 (25-40)	<0.001
**University affiliated hospitals**	489 (64.5%)	56 (70.0%)	0.61
**Role in the ICU**			<0.001
Nurses /Nurse assistants,	462 (60.4%)	45 (52.9%)	
Physicians,	231 (30.2%)	21 (24.7%)	
Fellows / Residents	6 (0.8%)	9 (10.6%)	
Other	66 (8.6%)	10 (11.8%)	
**Experience in the ICU, months**	90 (45-168)	9 (4-18)	<0.001
**Symptoms of depression**	196 (25.6%)	23 (27.1%)	0.88
**Symptoms of anxiety**	435 (56.9%)	57 (67.1%)	0.09
**HADS_A score**	7 (5-10)	8 (6-10)	0.24
**HADS_D score**	4 (2-7)	3 (2-7)	0.55
**Connor-Davidson Resilience Scale**	27 (23-31)	28 (22-31)	0.89
**Post-traumatic growth Inventory**	50 (32-64)	56 (41-67)	0.05

© 2025 Azoulay E et al. *JAMA Network Open*.

eFigure 1

HCPs' retrospective experience of the COVID-19 pandemic measured by VAS. Values for nursing staff (dark gray bars) and medical staff (light gray bars) all differ significantly (P < 0.01).

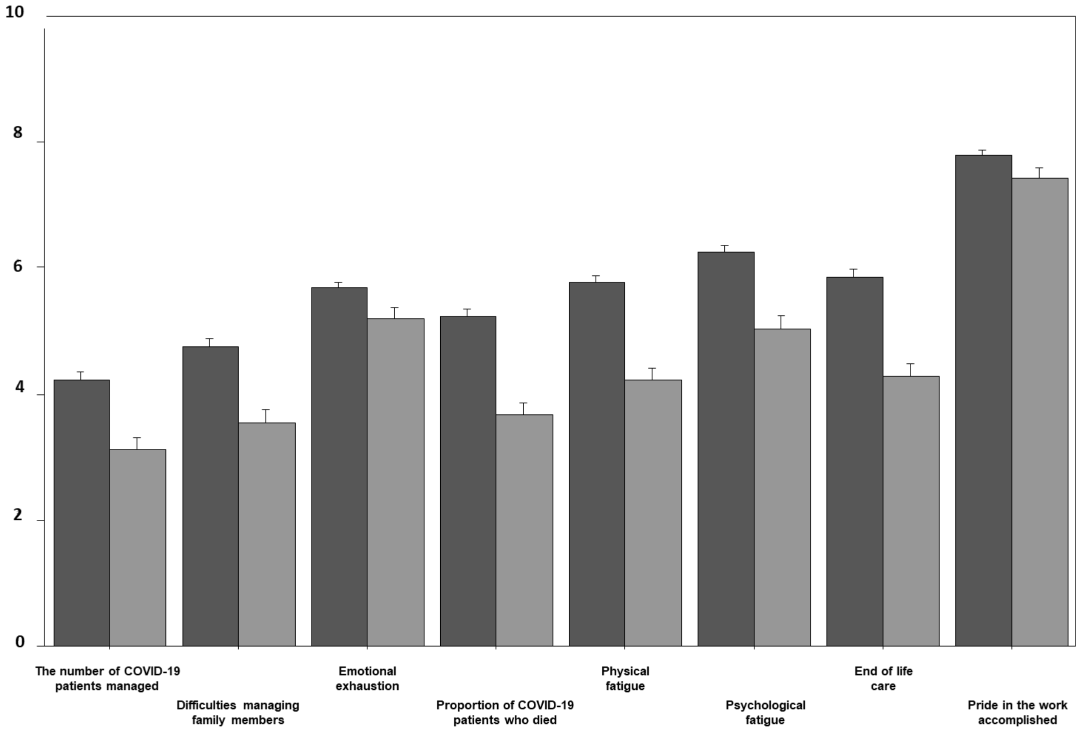

© 2025 Azoulay E et al. *JAMA Network Open*.

eFigure 2

Intensity of unidimensional measures (looking back to the pandemic, could you rate if the experience is still isolating, motivating, and makes you need a professional change). Ratings used visual analogic scales with two anchors: 0 (no symptom/lowest rating) and 10 (the most intense symptom/highest rating) in healthcare providers with and without symptoms of anxiety, depression, and resilience.

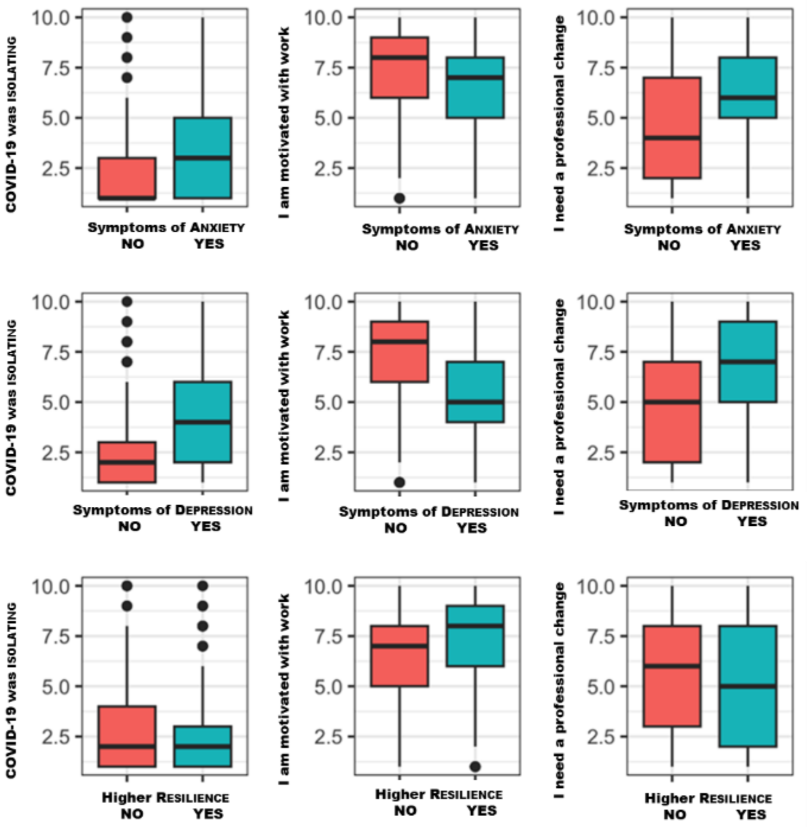

© 2025 Azoulay E et al. *JAMA Network Open*.

eAppendix

BURDENCOV 4th Survey

Prepared by the FAMIREA Group

Translated with the help of Chat GPT then carefully checked by the authors

Targeted participants: ICU healthcare professionals (nurses, aides, supervisors, doctors, interns, externs, physiotherapists, psychologists)

Introduction

This anonymous questionnaire (your email is not collected) follows three previous surveys conducted during the height of the COVID pandemic. These earlier surveys showed significant psychological impact among ICU staff. The 3rd survey explored resilience and led to a focus on Post-Traumatic Growth (PTG) — not only recovery from trauma but positive transformations.

This 4th survey aims to understand your post-COVID experience and your future outlook.

Mandatory question:

I agree that the data from this anonymous questionnaire may be used for scientific studies that will be published or presented at medical conferences.

☐ Yes (go to Question 2)

☐ No (go to Section 8 - Refusal to use your data)

Section 1: Personal Status

2. How are you doing professionally? (1 = Very good → 10 = Very bad)

3. How frightening do you find your professional situation?

4. How exhausting is your professional situation?

5. How frustrating is your professional situation?

6. Do you feel isolated at work?

7. How is your personal life compared to before COVID?

8. How have your working conditions evolved since 2020?

Section 2: Continued Impact of the Pandemic

9A. The number of COVID patients we had to care for during the epidemic

9B. The proportion of COVID patients who died

© 2025 Azoulay E et al. *JAMA Network Open*.

9C. Physical fatigue during the pandemic

9D. Mental fatigue during the pandemic

9E. End-of-life care management during the pandemic

9F. Conflicts within the ICU team

9G. Conflicts within the hospital

9H. Difficulties in relationships with patients' families

9I. What still affects me most is unrelated to my healthcare job

(Each on a scale of 1 = Not at all → 10 = Completely)

Section 3: Professional Recognition and Information Climate

10. In your opinion, are our professions more valued by society today?

11. Is the phenomenon of “infodemic” more frequent than before COVID?

12. Has family support in ICUs changed?

Options:

Yes, it has improved

Yes, it has worsened

No change over 4 years

Improved during the pandemic but now reverted

I wasn’t working in ICU during the pandemic

13. Have family visiting hours changed?

14. Are you satisfied with the way families are currently welcomed?

15. Compared to the pandemic, how would you rate your work engagement?

16. Are you still motivated/stimulated by your work?

© 2025 Azoulay E et al. *JAMA Network Open*.

Section 4: Motivational Sources & Pride

17. What are your main sources of motivation at work? (Multiple choices possible)

Examples: Diversity of care, teamwork, research projects, hierarchy relations, etc.

18. Are you proud of your work during COVID?

19. Are you proud of the work you do now?

(1 = Not at all proud → 10 = Extremely proud)

Section 5: Change & Personal Development

20. Do you feel the need for a professional change?

21. If yes, what kind? (Multiple answers possible)

Examples: Change department, go private, leave the profession, etc.

22. Do you feel the need for personal change?

23. If yes, what kind? (Multiple answers possible)

Examples: Invest more in family/social life, rest more, enjoy hobbies, etc.

Section 6: Support & Psychological Help

24. Who do you feel supported by? (Multiple answers possible)

Options: colleagues, hierarchy, institution, friends, family, government, society, none

25. Have you consulted a psychologist or psychiatrist?

Options:

Yes (in the department, hospital, outside)

No (no time / not needed)

Section 7: Daily Habits & Demographics

26. Daily time spent on:

© 2025 Azoulay E et al. *JAMA Network Open*.

Watching TV

Using social media

On your phone

Reading newspapers

Reading books

27. What motivates you today, during both hard times and stability? (Open keywords)
28. Gender: Male / Female / Prefer not to say
29. Age
30. Type of facility: General Hospital / University Hospital / Private
31. Has anything major happened to you personally since the pandemic?
32. City of residence
33. Job title / status (nurse, physician, intern, etc.)
34. Months worked in ICU
35. ICU experience during each half-year since 2020
36. Most difficult half-year personally or professionally

Section 8: Coping & Substance Use

37. Have you used psychotropics (anxiolytics, sleeping pills, antidepressants)?
38. If yes, who prescribed them? (Multiple options)
39. Excessive alcohol consumption?
40. Cannabis use?
41. Recreational drug use?

Each with checkboxes per semester and "still today"

Section 9: COVID & Mental Health

© 2025 Azoulay E et al. *JAMA Network Open*.

42. Have you had COVID?

43. What about people close to you?

44. Have you been vaccinated in 2023?

45. Have you consulted a psychologist/psychiatrist during specific periods?

© 2025 Azoulay E et al. *JAMA Network Open*.

HADS (Hospital Anxiety and Depression Scale)

Hospital Anxiety and Depression Scale

Directions: Please choose the answer that best describes how you have been feeling during the LAST WEEK. Don't think too long over your replies; your immediate answer is best.

A. I feel tense or 'wound up':<br>3. Most of the time<br>2. A lot of the time<br>1. From time to time, occasionally<br>0. Not at all	D. I still enjoy the things I used to enjoy:<br>0. Definitely as much<br>1. Not quite so much<br>2. Only a little<br>3. Hardly at all
A. I get a sort of frightened feeling as if something awful is about to happen:<br>3. Very definitely and quite badly<br>2. Yes, but not too badly<br>1. A little, but it doesn't worry me<br>0. Not at all	D. I can laugh and see the funny side of things:<br>0. As much as I always could<br>1. Not quite so much now<br>2. Definitely not so much now<br>3. Not at all
A. Worrying thoughts go through my mind:<br>3. A great deal of the time<br>2. A lot of the time<br>1. From time to time, but not too often<br>0. Only occasionally	D. I feel cheerful:<br>3. Not at all<br>2. Not often<br>1. Sometimes<br>0. Most of the time
A. I can sit at ease and feel relaxed:<br>0. Definitely<br>1. Usually<br>2. Not often<br>3. Not at all	D. I feel as if I am slowed down:<br>3. Nearly all the time<br>2. Very often<br>1. Sometimes<br>0. Not at all
A. I get a sort of frightened feeling like 'butterflies' in the stomach:<br>0. Not at all<br>1. Occasionally<br>2. Quite Often<br>3. Very Often	D. I have lost interest in my appearance:<br>3. Definitely<br>2. I don't take as much care as I should<br>1. I may not take quite as much care<br>0. I take just as much care as ever
A. I feel restless as if I have to be on the move:<br>3. Very much indeed<br>2. Quite a lot<br>1. Not very much<br>0. Not at all	D. I look forward with enjoyment to things:<br>0. As much as I ever did<br>1. Rather less than I used to<br>2. Definitely less than I used to<br>3. Hardly at all
A. I get sudden feelings of panic:<br>3. Very often indeed<br>2. Quite often<br>1. Not very often<br>0. Not at all	D. I can enjoy a good book or radio or TV program:<br>0. Often<br>1. Sometimes<br>2. Not often<br>3. Very seldom

© 2025 Azoulay E et al. *JAMA Network Open*.

Post-Traumatic Growth Inventory (PTGI)

Post Traumatic Growth Inventory

Client Name:________________________________ Today's Date:_______________

Indicate for each of the statements below the degree to which this change occurred in your life as a result of the crisis/disaster, using the following scale.

0 = I did not experience this change as a result of my crisis.
1 = I experienced this change to a very small degree as a result of my crisis.
2 = I experienced this change to a small degree as a result of my crisis.
3 = I experienced this change to a moderate degree as a result of my crisis.
4 = I experienced this change to a great degree as a result of my crisis.
5 = I experienced this change to a very great degree as a result of my crisis.

Possible Areas of Growth and Change	0	1	2	3	4	5
1. I changed my priorities about what is important in life.						
2. I have a greater appreciation for the value of my own life.						
3. I developed new interests.						
4. I have a greater feeling of self-reliance.						
5. I have a better understanding of spiritual matters.						
6. I more clearly see that I can count on people in times of trouble. Text						
7. I established a new path for my life.						
8. I have a greater sense of closeness with others.						
9. I am more willing to express my emotions.						
10. I know better that I can handle difficulties.						
11. I am able to do better things with my life.						
12. I am better able to accept the way things work out.						
13. I can better appreciate each day.						
14. New opportunities are available which wouldn't have been otherwise.						
15. I have more compassion for others.						
16. I put more effort into my relationships.						
17. I am more likely to try to change things which need changing.						
18. I have a stronger religious faith.						
19. I discovered that I'm stronger than I thought I was.						
20. I learned a great deal about how wonderful people are.						
21. I better accept needing others.						

© 2025 Azoulay E et al. *JAMA Network Open*.

CD-RISC 10 (Resilience Scale)

Please indicate to what extent you agree or disagree with each of the following statements. Try to answer spontaneously.

<u>Each item is rated on the following scale:</u>

0 = Not true at all

1 = Rarely true

2 = Sometimes true

3 = Often true

4 = True nearly all the time

Items

1. I am able to adapt when changes occur.
2. I can deal with whatever comes my way.
3. I try to see the humorous side of things when I am faced with problems.
4. Dealing with stress can make me stronger.
5. I tend to bounce back after illness, injury, or other hardships.
6. I believe I can achieve my goals, even if there are obstacles.
7. Under pressure, I stay focused and think clearly.
8. I am not easily discouraged by failure.
9. I think of myself as a strong person when facing life's challenges and difficulties.
10. I am able to handle unpleasant or painful feelings such as sadness, fear, or anger.

© 2025 Azoulay E et al. *JAMA Network Open*.

Final Note

THANK YOU FOR YOUR PARTICIPATION

If you do not wish your data to be used, you cannot complete the questionnaire.

Let me know if you want this content:

As a downloadable Word document or PDF

Formatted into a Google Form

Or need a summary or focus on a particular section (e.g., HADS or PTGI scales)

I'm happy to help with formatting or analysis as well.

© 2025 Azoulay E et al. *JAMA Network Open*.
