# Supplementary material for: Posttraumatic Growth in Intensive Care Unit Health Care Professionals After COVID-19
Source: JAMA Netw Open. 2025 Aug 25;8(8):e2527443. doi: 10.1001/jamanetworkopen.2025.27443 (PMC12379108; doi:10.1001/jamanetworkopen.2025.27443)
Supplement: Supplement 2. — Data Sharing Statement [file jamanetwopen-e2527443-s002.pdf]

Data Sharing Statement

Azoulay. Posttraumatic Growth in Intensive Care Unit Health Care Professionals After COVID-19. *JAMA Netw Open*. Published August 25, 2025. doi:10.1001/jamanetworkopen.2025.27443

Data
Data available: Yes
Data types: Data (not involving human participants)
How to access data: healthcare providers
When available: With publication

Supporting Documents
Document types: None

Additional Information
Who can access the data: Dr Elie Azoulay
Types of analyses: any
Mechanisms of data availability: invest support
